# Investigating the mechanisms of small extracellular vesicles in cardiovascular disease using the living myocardial slice platform

**DOI:** 10.3389/fcvm.2025.1697099

**Published:** 2025-11-12

**Authors:** Fani Koutentaki, Laura Nicastro, Richard J. R. Kelwick, Paul S. Freemont, Cesare M. Terracciano

**Affiliations:** 1 National Heart and Lung Institute, Imperial College London, London, United Kingdom; 2 Section of Structural and Synthetic Biology, Department of Infectious Disease, Imperial College London, London, United Kingdom

**Keywords:** cardiovascular disease, extracellular vesicles, living myocardial slices, small extracellular vesicles, mechanisms, cell-cell crosstalk

## Abstract

Cardiovascular disease remains the leading cause of death worldwide. Extracellular vesicles (EVs) play a regulatory role in homeostasis, associated with their contribution to cell-cell communication. Recently, it has been confirmed that they also regulate the progression of cardiovascular disease. Specifically, myocardial injury induces an increase in the secretion of small extracellular vesicles (sEVs), both in the cardiac microenvironment and peripheral circulation. Small extracellular vesicles (sEVs) are lipid bilayer particles within the size range of 35–200 nm and are secreted by all cell types. Their high content of bioactive cargo—primarily miRNA—is altered in response to external stimuli, leading to behavioral changes of the recipient cells. In the context of cardiovascular disease, this change leads to acute and long term functional, structural, and biochemical effects on the myocardium. However, the mechanism behind the altered sEVs secretion and their changes in content in the context of cardiovascular disease is yet to be determined. That is partially due to the challenges associated with the isolation of cardiac-derived sEVs, which are essential for the investigation of the mechanisms behind cardiovascular disease progression. Living myocardial slices (LMS) provide an ideal platform for the isolation and investigation of sEVs function in the myocardium. Indeed, LMS not only maintain the cellular complexity and architecture of the native adult myocardium but can also be cultured over days/weeks without significant alterations in cardiac function, making them a reliable model for sEVs isolation and characterization at multiple timepoints. This review aims to summarize recent findings on the effect of sEVs on the onset and progression of cardiovascular disease and to discuss different methods for their isolation from LMSs and the investigation of their functional, structural, and biochemical effect on the myocardium.

## 1 Introduction

Cardiovascular disease (CVD) is amongst the leading causes of mortality globally (1). Common CVDs include arrhythmia, coronary artery diseases (CAD), valve disease, hypertrophy, stroke, congenital heart disease, and heart failure. In the UK, 7.6 million people are living with heart and circulatory diseases, and 175,000 deaths are reported annually. The cost of CVDs in the UK reaches the substantial amount of 12 billion pounds annually (2). The ageing population and the increase of risk factors associated with CVD, including diabetes, obesity, pulmonary hypertension, sedentary lifestyle, and stress, are causing a rise in CVD prevalence (3, 4). Effective treatment options are limited and involve surgical interventions—heart transplant, valve repair, coronary artery bypass graft (CABG) surgery, primary percutaneous coronary intervention (PPCI)—or medical therapy for the alleviation of symptoms (5, 6). Despite the recent advances in the research field, a lot of gaps still remain in the knowledge behind the molecular mechanisms that induce the onset and progression of CVD, while there is also lack of relevant *in vivo* and *in vitro* CVD models.

Extracellular vesicles (EVs)—particularly small EVs (sEVs)—have received attention in the cardiovascular field of research, due to their contribution to cell-cell communication. sEVs are known to be enriched with bioactive molecules and are released by donor cells to be taken up by target cells, where they deliver their cargo. Delivery of molecular contents by sEVs can influence gene expression and cell behavior of recipient cells both in health and disease. Their ability to translocate through the extracellular space and to deliver bioactive molecules in a targeted manner highlights their high potential in the field of therapeutics as tools for drug delivery (7). The shift in their content during disease conditions can provide insight in the presence of biomarkers in specific diseases, allowing their utilization as diagnostic or prognostic tools (7–9). Even though the sEV field is growing, a lot remains to be understood regarding the mechanisms behind the changes in sEV secretion and uptake and their participation in CVD, in part because of the lack of relevant experimental models. Thus, it is of high importance to develop relevant translational models for the investigation of the mechanistic role of sEVs in CVD.

The living myocardial slice (LMS) model is an ultra-thin layer of myocardium that maintains the multicellularity and extracellular matrix of the adult cardiac tissue, hence preserving the structural and electrophysiological properties of the tissue, whilst continuously secreting sEVs (10). Moreover, LMS can be manipulated to recreate disease models *in vitro* and investigate sEV dynamics at different timepoints of disease.

This review aims to summarize the recent advances on the effect of sEVs on the onset and progression of CVD, as well as to introduce and discuss the advantages of the LMS model as a powerful tool for the investigation of the mechanistic role of sEVs in CVD. Finally, it provides detailed methodology for cardiac sEV isolation from LMSs, techniques for cardiac sEV characterisation, and for the investigation of their functional, structural, and biochemical effects on CVDs using the human LMS model.

## 2 Biology of small extracellular vesicles

### 2.1 sEV definition and content

Extracellular vesicles (EVs) are heterogeneous lipid bilayer nanoparticles of several subtypes, sizes, and origins that contribute to intercellular communication in homeostasis and disease (11). The subtypes differ in terms of their size and biogenesis pathway and include small extracellular vesicles (sEVs) or exosomes, microvesicles, and apoptotic bodies. sEVs range from 35 to 200 nm, they are of endocytic origin and are released by all eukaryotic cell types (7). sEVs can be detected in body fluids, including sweat, blood, saliva, and urine (12).

Within their lipid bilayer, EVs carry bioactive molecules including DNA, proteins, lipids, and RNA, which upon uptake by recipient cells influence their behavior and mediate intercellular communication. microRNA (miRNA)—non-coding RNA that regulates gene expression post-transcriptionally—is the most abundant type of RNA contained in sEVs and has been established as a strong functional mediator in cellular communication (13). Other than their size, sEVs can be distinguished from other microvesicles by the presence of sEV-specific markers, which include the ESCRT complex, MVB-related protein Alix, heat-shock proteins, tumor susceptibility gene 101 protein (TSG101), clathrin, and transmembrane proteins of the tetraspanin family (CD9, CD63, CD81) (12).

### 2.2 sEV biogenesis pathway

**Figure 1:**
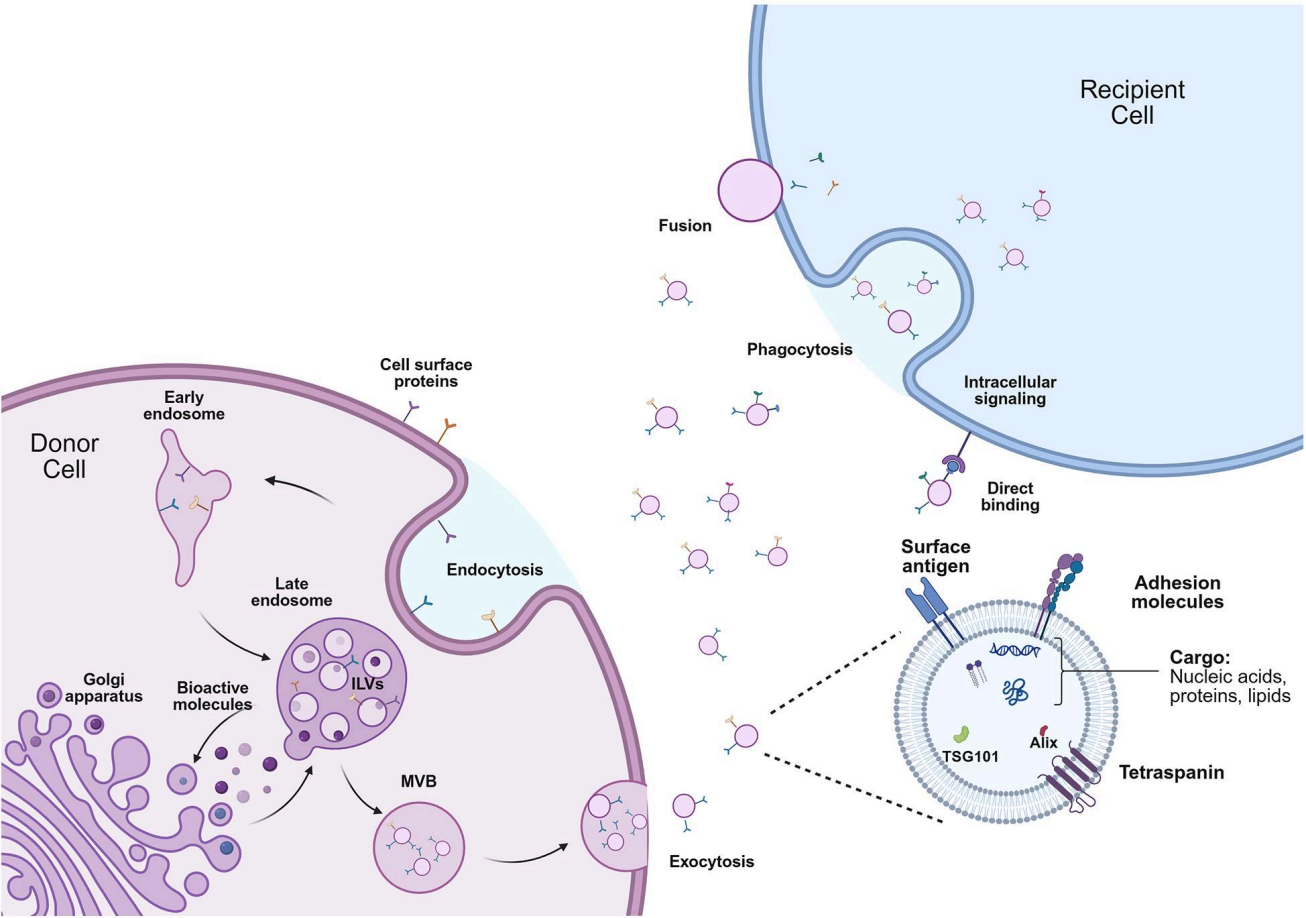
Biogenesis pathway of sEVs. sEVs are formed by the initial inward budding of the plasma membrane, generating the early endosomes. Bioactive molecules in the intracellular space are enclosed into late endosomes, forming intraluminal vesicles (ILVs) and packaged in multivesicular bodies (MVBs). The sEVs are released in the extracellular space by exocytosis to reach the recipient cell. Uptake mechanisms include phagocytosis, fusion, or direct binding with plasma membrane receptors. Adapted with permission from “Biogenesis and Cellular uptake of EVs” by Yuanwang Jia, Li Yu, Tieliang Ma, Wenrong Xu, Hui Qian, Yaoxiang Sun and Hui Shi, licensed under CC BY 4.0.

The sEV biogenesis pathway is initiated by the inward budding of the cell membrane to form early endosomes (Figure 1). Bioactive molecules in the intracellular space are enclosed in the lipid membrane and then mature into late endosomes. Late endosomes form intraluminal vesicles (ILVs)—sEV precursors—through inward invagination and are packaged into multivesicular bodies (MVBs) (14). The molecular content and ubiquitination status of MVBs determines their fate, which is either degradation by lysosomes or fusion into the cell membrane to exocytose the sEVs in the extracellular space (15). The mechanisms involved in these processes are very complex and outside of the scope of this review. However, there are some excellent reviews where these mechanisms are explored further (12, 16–18). Following detachment from the membrane, the sEVs retain their unique structure, including transmembrane proteins of the tetraspanin family (CD9, CD63, and CD81), which facilitate the loading of bioactive molecules in sEVs and play a crucial role in uptake mechanisms (12, 15–18).

### 2.3 sEV uptake mechanisms

Once released from the cell, sEVs travel through the extracellular space to enter circulation and reach other tissues or to be taken up by target cells within the same tissue (19). Uptake mechanisms are achieved by the unique composition of receptors and antigens found in the lipid bilayer of sEVs (14) (Figure 1). Specifically, sEVs can directly fuse to the plasma membrane, delivering their content in the intracellular space of the recipient cell or directly bind to surface receptors and initiate intracellular signaling (20). In many cases, sEVs are taken up by the cell via endocytosis or phagocytosis, transferred in endosomes, and re-released in the intracellular space to secrete their contents or to undergo lysosomal degradation (21, 22).

### 2.4 sEV role in homeostasis and alterations in CVD

The miRNA content that is received by the recipient cell can affect its gene expression profile and in turn its phenotype. sEV content can differ depending on the cell type of origin and the state of the tissue releasing it—homeostatic or diseased. Under normal conditions, sEVs participate in cell-cell crosstalk to regulate major pathways, including proliferation, inflammation, angiogenesis, fibrosis, and apoptosis, to maintain homeostasis. Under stressful conditions or upon injury, the concentration and content of sEVs changes to impact the recipient cell's behavior accordingly as a response to the external stimulus. sEVs secreted by different cardiac cell types exert diverse influences on the recipient cells, due to their differential bioactive molecule content. These effects are structural, molecular, and eventually functional, and can vary depending on disease severity and stage. It is essential to investigate these effects to understand the role of sEVs on CVD progression (23).

## 3 sEV functional, structural, and biochemical alterations in CVD

### 3.1 sEVs increase in circulation in CVD

The role of sEVs in CVD progression is of high interest due to their participation in intercellular communication. The myocardium is comprised primarily of cardiomyocytes (CMs), cardiac fibroblasts (CFs), endothelial cells (ECs), vascular smooth muscle cells, and immune cells (24). The continuous secretion and uptake of sEVs leads to the interaction of the cardiac cell types and the influence of their behavior for the maintenance of homeostasis and for response to stressful conditions (25, 26). Depending on the cell type of origin and the conditions upon secretion, the sEV concentration and miRNA content will differ. Emanueli et al. demonstrated that following CABG surgery, the concentration of sEVs of cardiac origin and onto peripheral circulation rises and that their cardiac miRNA content is increased (27). It has also been confirmed that cardiac miRNA levels are increased in the plasma of acute myocardial infarction (MI) patients (9). More recently, this was also demonstrated in patients of ST-elevation myocardial infarction (STEMI)—the most severe type of acute MI. Following reperfusion via PPCI, the plasma of 42 patients was collected for circulating sEVs' isolation. Quantification of sEVs showed their increase in STEMI patients and in patients subjected to late reperfusion. Further investigations in sEV content and marker expression can be informative regarding their role in disease severity and progression. In this same study, it was observed that the sEV expression of platelet-specific markers showed a positive correlation with ejection fraction and a negative correlation with the degree of microvascular obstruction, evaluated with cardiac magnetic resonance (28). It is also interesting that depending on the clinical setting, the effect of sEVs may differ. This was demonstrated in a study investigating the effect of sEVs derived from the serum of diabetic mice on non-diabetic mouse models subjected to MI reperfusion (MI/R) injury. Specifically, the intramyocardial injection of sEVs aggravated the damage of MI/R, indicated by diminished functional recovery and an increase in scar size and CM death (29).

Work has also been performed for larger EV types. A study exposed Langendorff-perfused rat hearts to ischemia and reperfusion and isolated microvesicles from the coronary perfusate to investigate their response to the injury. Quantification of an established EV marker confirmed an increase in the concentration of microvesicles (30). This was also demonstrated in mice subjected to acute MI by coronary artery ligation. In this study, EVs were isolated from LV tissue and large and small EVs were quantified separately, to confirm a higher concentration for the large EVs. These findings were also confirmed in clinical samples collected from patients that had undergone aortic valve replacement (31).

### 3.2 sEV content shifts in CVD

#### 3.2.1 sEVs released by CMs

**Figure 2:**
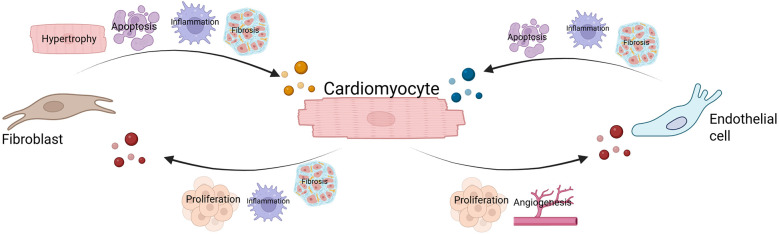
sEV-mediated regulation of biological processes. sEVs are produced by all cardiac cell types. Their content mediates intercellular communication, which results in the influence of several pathways, including the proliferation, inflammation, fibrosis, apoptosis, and angiogenesis. Arrows indicate the direction of sEVs released from cardiomyocytes (red), endothelial cells (blue), and fibroblasts (orange).

The altered sEV release and protein content from CMs in response to CVD can affect pathways of angiogenesis, apoptosis, inflammation, proliferation, and fibrosis (Figure 2). Gupta et al. first showed that CMs release sEVs*.* The aim of the study was to understand the secretion pathway of heat shock protein 60 (HSP60), which is a protein that protects tissues during injury. Their findings demonstrated that adult CMs secrete HSP60 via exosomes and that hypoxia-reoxygenation injury (HRI) increased the secretion of HSP60-containing exosomes by CMs by three times compared to healthy conditions (32).

Ever since, others have shown that following ischemia-reperfusion injury (IRI), CMs secrete sEVs that play a role in cell-cell crosstalk. Chen et al. demonstrated that in a murine model of HRI, the triggering of the eNOS pathway—cardioprotective for ECs—can occur following the uptake of CM-derived sEVs by ECs (33). Importantly, the impact of miRNAs carried by CM-derived sEVs on other cardiac cell types has become a significant area of research, with many of these miRNAs now recognized as cardiac-specific. Anselmo et al. (34) recently demonstrated that the surface antigen CD172a is specifically expressed on EVs secreted by cardiomyocytes (CMs), enabling the identification of the cellular origin of sEVs. These sEVs were found to be enriched with cardiac-specific miRNAs and were produced in response to cellular stress triggered by hypoxic conditions. Additionally, the study revealed that patients with aortic stenosis who underwent transcatheter aortic valve replacement and had elevated levels of CD172a+ EVs in their peripheral blood showed improved survival outcomes (34).

The miRNAs that are released from CMs can have a significant role in disease progression. Yang et al. (35) demonstrated that hypoxic conditions lead to elevated levels of miR-208a in rat CMs. Furthermore, when exosomes from these hypoxic CMs were co-cultured with CFs, the CFs showed increased levels of miR-208a, which stimulated their proliferation and myofibroblast activation, thereby aggravating fibrosis (35). In terms of CM–EC communication, sEVs released from glucose-deprived CMs were found to be enriched in miR-17, miR-20, miR-23a, miR-23b, miR-30c, and miR-126-3p. Upon uptake by ECs, these miRNAs enhanced EC proliferation and promoted angiogenesis (36).

#### 3.2.2 sEVs released by CFs

CFs play a key role in triggering and sustaining inflammation and tissue remodeling after cardiac injury. As part of this process, they secrete sEVs enriched with miRNAs that can modulate the behavior of other cell types. Bang et al. demonstrated that exosomes isolated from CFs of mice subjected to transverse aortic constriction, when applied to cultured CMs, induced hypertrophic response in the CMs. Further analysis confirmed that CMs internalised these CF-derived exosomes, which delivered miR-21_3p - a CF-specific miRNA known to drive stress-induced hypertrophy (106).

Cosme et al. conducted a study to assess how sEVs from CFs exposed to HRI affect CM viability. Proteomic comparisons between sEVs from control and hypoxic CFs revealed 71 proteins with significantly increased levels and 73 with decreased levels, many of which were associated with fibrotic and tissue remodeling pathways. The study also demonstrated that the impact of CF-derived sEVs on CMs varied depending on the state of the recipient cells upon administration. When applied to healthy CMs, the sEVs enhanced cell survival, whereas their application after HRI led to diminished viability (37).

Another study demonstrated that CF-derived sEVs released during HRI contain miR-133a. This miRNA was found to inhibit the expression of embryonic lethal abnormal vision like 1 (ELAVL1), a protein known for its pro-inflammatory role. By downregulating ELAVL1, miR-133a contributed to decreased inflammation and reduced apoptosis in CMs (38).

#### 3.2.3 sEVs released by ECs

ECs are key components of blood vessels, playing a crucial role in mediating communication between the bloodstream and surrounding tissues. During ischemic events, blood flow is restricted, leading to EC injury and the subsequent release of sEVs (24). In one study, sEVs derived from stem cell-derived ECs exposed to hypoxic conditions were found to suppress apoptosis and promote angiogenesis. This, in turn, enhanced CM survival and contractile function in a human IRI heart-on-a-chip model (39).

A study examining cultured human ECs found that hypoxic conditions alter the proteomic composition of their secreted EVs. Notably, there was an increase in proteins such as lysyl oxidase-like 2 (LOXL2), fibronectin, and collagen, suggesting that these EVs play a role in promoting inflammation, with a particular emphasis on tissue remodeling and fibrotic processes (40).

Davidson et al. demonstrated that co-culturing human umbilical vein ECs with CMs prior to HRI significantly enhanced CM survival. This protective effect was attributed to the exosomal content released by ECs, which activated the ERK1/2 MAPK signaling pathway in the CMs (41). The existing literature is summarized in Table 1.

**Table 1:** Overview of cardiac miRNA effects on proliferation, apoptosis, inflammation, angiogenesis, and fibrosis pathways.

Origin	Bioactive molecule	Target gene/pathway	Model	Effect	Reference
Mouse CMs	HSP60	Stress response pathway	*In vitro* CM culture	HSP60 is released via exosomes; its release triples after HRI	Gupta & Knowlton (32)
Mouse CMs	Various proteins & miRs	Stress, fibrosis, remodeling	Murine IRI model	CMs produce sEVs in response to IRI	Chen et al. (33)
Mouse CMs	CD172a+ sEVs (cardiac miRs)	Hypoxia response	Mouse model + human patients (AS)	CD172a+ sEVs increase under hypoxia; higher levels correlate with higher survival in AS patients	Anselmo et al. (34)
Rat CMs → CFs	miR-208a	Fibrosis, proliferation	Hypoxic CMs co-cultured with CFs	miR-208a transferred to CFs promotes proliferation and myofibroblast activation	Yang et al. (35)
Mouse CMs → ECs	miR-17, 20, 23a/b, 30c, 126-3p	Angiogenesis	Glucose-deprived CMs, EC co-culture	Promotes EC proliferation and angiogenesis	Garcia et al. (36)
Mouse CFs	rno-miR-21-3p	Hypertrophy pathway	CM-CF 2D co-culture	CF-derived exosomes containing miR-21-3p increased CM hypertrophy	Bang et al., (106)
Mouse CFs	Various proteins (proteomics)	Remodeling and fibrosis	Hypoxic CFs → CMs	71 proteins ↑, 73 ↓ post-HRI; time-dependent effect on CM survivability	Cosme et al. (37)
Mouse CFs	miR-133a	ELAVL1 (inflammation/apoptosis)	Hypoxic CFs → CMs	Suppressed ELAVL1, reduced inflammation and apoptosis	Liu et al. (38)
Human ECs	Unknown miR	Apoptosis, angiogenesis	Human IRI heart-on-a-chip	Increased CM survival and contractility via inhibition of apoptosis and promotion of angiogenesis	Yadid et al. (39)
Human ECs	LOXL2, fibronectin, collagen	Inflammation, tissue remodeling	Hypoxic EC culture	Proteomic changes support role in remodeling/fibrosis	de Jong et al. (40)
Human ECs	Exosomal proteins	ERK1/2 MAPK signaling	EC-CM co-culture prior to HRI	Increased CM viability due to EC exosome-induced ERK1/2 activation	Davidson et al. (41)

HSP, heat-shock protein; CM, cardiomyocyte; HRI, hypoxia-reoxygenation injury; IRI, ischemia-reperfusion injury; sEVs, small extracellular vesicles; miR, microRNA; EC, endothelial cell; CF, cardiac fibroblasts; ELAV1, embryonic lethal abnormal vision like 1; LOXL2, lysyl oxidase-like 2.

### 3.3 Gaps in the sEV field

Despite the extensive literature in the role of sEVs in CVDs, there are still fundamental gaps in the field, both in the context of homeostasis and disease, that do not allow the understanding of sEVs' role in disease progression and prevention. Specifically, there are questions regarding the basic biology of EVs, due to the high heterogeneity of the molecular content and structure (12). Moreover, even though there has been an increased understanding behind the several sEV uptake mechanisms, which include surface receptor interactions with the recipient cell and by internalization of the sEVs by phagocytosis or clathrin-mediated uptake, it is still not well understood how these mechanisms change in response to disease. It is of high interest to establish the mechanistic shift of sEV secretion, uptake, and content change in CVD. The remaining questions are due to the challenges in studying sEVs and the lack of *in vivo* and *in vitro* experimental models that can overcome them (11).

## 4 Challenges in studying cardiac-derived sEVs

### 4.1 sEV isolation and characterisation

There are many factors that make sEV research challenging. Firstly, sEVs are nanosized particles that are difficult to isolate and quantify. Their small size obstructs the accurate determination of their identity and origin. Another challenge is their heterogeneity. EVs are a large population of particles of multiple subtypes, sizes, and origins (11). Even though each population expresses specific markers and is within specific size ranges, there is still an overlap. For example, sEVs express the tetraspanin proteins, but they are also expressed by microvesicles at lower levels (42). Additionally, the size limits are not strict. sEVs range from 30–200 nm, while microvesicles from 100–1,000 nm. Thus, it is essential that appropriate techniques are used to accurately isolate and characterize different EV populations, while also separating them from other non-vesicular populations (43). Finally, the molecular heterogeneity of sEVs is also a challenge, especially in the context of disease. The temporal dynamics of sEV secretion and content change of different cell types but also within the same at the different stages of disease progression make their characterisation more challenging. Recently, sEV separation and characterisation technologies have progressed to improve isolation purity and yield, however it still remains a challenge to achieve both quantity and specificity (11). These challenges can be minimized by following the minimal information for studies of extracellular vesicles (MISEV), which proposes strategies for the isolation and characterisation of EVs, depending on the source, type, and general characteristics (11, 43).

### 4.2 Lack of relevant experimental models

The gaps in understanding the role of sEVs in CVD are in part due to the lack of appropriate experimental models. When studying the functional role of sEVs, the choice of model is crucial, as it directly influences the quality, relevance, and interpretability of the sEVs produced. In the context of CVD, it is particularly important to be able to obtain pure cardiac-derived sEVs specifically. Consequently, the more physiologically relevant the model, the more representative and informative the sEVs obtained will be. There is a critical need for experimental systems that not only reflect the pathophysiology of CVD but also enable the isolation and investigation of myocardium specific-derived sEVs, to uncover their role in disease onset and progression.

Several experimental models have been employed to study sEVs in CVD, each showing defects in terms of physiological relevance and feasibility. Studies that use monolayers of cells fail to include the multicellular and extracellular matrix components of the cardiac tissue, making them minimalistic models (44). Consequently, the sEVs derived from these models are of lower numbers and from limited cell types. It is also important to consider the origin of cellular models. Many studies use cells that are derived from animal neonatal stem cells or induced pluripotent stem cells (iPSCs), which show substantial differences from adult human cardiac cells in terms of metabolism and Ca^2+^ handling and sEV secretion and content (45).

Engineered heart tissue, organoids, or organ-on-a-chip models are three dimensional (3D), hence eliminating the limitations of monolayers of cells. They include more cell types and extracellular components, thus incorporating an increased complexity in the model (46). However, most of these models can only be recreated using neonatal CMs and stem cell-derived CMs, who exhibit fetal maturity. Moreover, these models lack the biomimetic environment that is crucial for maintenance of the myocardium *in vivo*, such as mechanical load and electrical stimulation. These factors play a significant role in cardiac cell maturation and function and will reflect the profile of the sEVs they produce (47).

The performance of *in vivo* studies using animal models adds an increased complexity in the investigation, due to the involvement of inflammation and the participation and response of other organs, affecting the applicability of the results and subsequent conclusions (44). The secretion of sEVs from other organs increases the heterogeneity of the sEV populations isolated, complicating characterisation. Animal models also display differences in cardiac size and physiology, compared to humans, while CVD phenotypes may also vary (48). This makes the findings less relevant and translational.

## 5 The living myocardial slice model

### 5.1 Living myocardial slice: the model of intermediate complexity

The living myocardial slice (LMS) model is an ultra-thin layer of myocardium (300–350 μm). It is considered a model of intermediate complexity, as it maintains the multicellular and extracellular matrix components of adult cardiac tissue, while avoiding the high complexity of animal models. This suggests that they preserve the electrophysiological properties of the native tissue, as they represent its functional, structural, mechanical, and biochemical properties (10).

**Figure 3:**
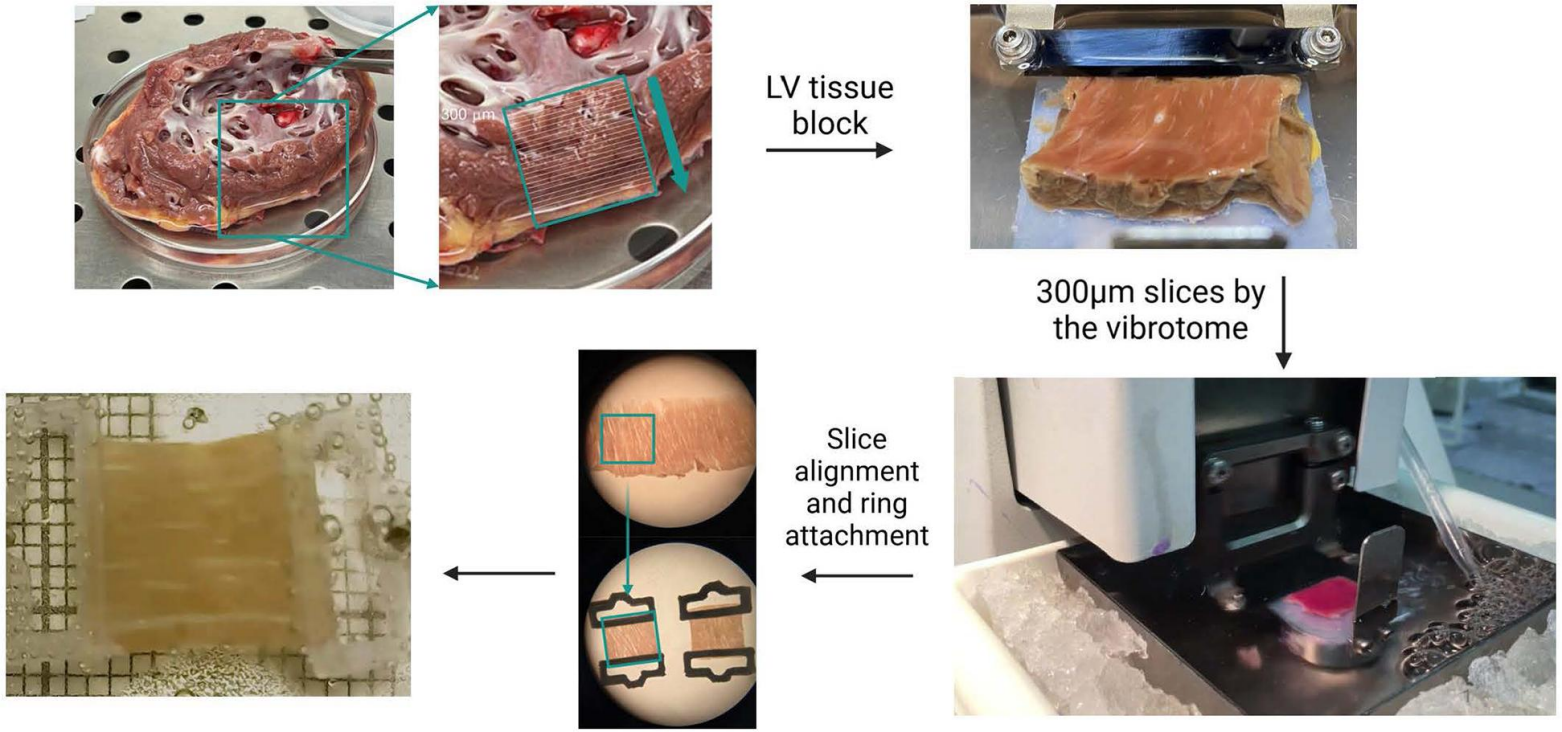
Generation process of the human LMS model. The human donor heart is dissected to retrieve an LV block of 1 cm^3^. The vibratome precision-cuts the block to obtain layers of 300 μm thickness. The slice is aligned under the microscope for the positioning of the PLA rings perpendicular to the muscle fibers. The LMS is ready to culture.

The LMS model can be prepared from several species, including rat, rabbit, lamb, pig, and human, as previously described (10, 44, 49, 105). The method of human LMS preparation involves different steps (Figure 3). Briefly, following heart explant and dissection, a left ventricular (LV) block of 1 cm^3^ is placed in a high-precision vibratome, which precision-cuts the LV in 300 μm thick layers. To generate the LMS, each layer is trimmed to achieve 1 cm^2^ slices, followed by the attachment of polyactic acid (PLA) rings perpendicular to the fibers' direction to not hinder contraction.

LMS can then be cultured in biomimetic conditions using isometric chambers. Specifically, the LMSs are placed in customized stretchers that allow application of mechanical preload by stretching the slices at different sarcomere lengths. In that way, we provide a degree of mechanical load to closely recapitulate mechanical preload. The chambers can then be supplemented with pure O_2_ or 95% CO_2_ and media rich in hormones, nutrients, and salts, which is constantly circulated through the chamber. Finally, electrical stimulation is delivered throughout the culture period (10, 44). These culture conditions have been optimised to allow for the long-term survival of the LMS model, as its thin structure allows for sufficient O_2_ and metabolite diffusion (50). Under these conditions the LMS model continues to secrete sEVs throughout the entire culture duration, that can be later isolated from the media for further investigation.

### 5.2 Using the LMS to model CVD

The LMS model can be used to model CVD and investigate sEV dynamics at different stages of disease, allowing its use as a platform to investigate mechanisms of biological processes. The use of diseased or failing hearts will allow preservation of the pathological characteristics of the tissue, as they similarly maintain the functional and structural properties of the disease. Alternatively, the LMS model can be manipulated to recapitulate CVD, by either altering the culture conditions or artificially recreating the disease condition, depending on the desired phenotype (44, 51). In any case, it is important to consider that a single LV piece can generate a sufficient number of LMSs to allow for reproducible experiments and potential replacement of animal models.

The LMS model has been used to simulate different CVD. For example, manipulating the amount of preload during culture by overstretching the LMS using the stretchers results in the pathological effects observed in heart failure (51). Moreover, procedures can be performed on the LMSs to induce injury. Dries et al. used rat LMSs to induce cryoinjury and observe the effects after culture (52). Ischemia-reperfusion or hypoxia-reoxygenation injury can be induced by the manipulation of O_2_ levels in culture (53, 54).

### 5.3 Limitations of the LMS model

Despite the numerous advantages of the LMS model, there are a few points to consider. Firstly, the LMS model preparation requires practice of several months before obtaining appropriate and viable samples for experimentation. This is primarily due to the skills required for the dissection and slice trimming technique, appropriate positioning of the PLA rings, and careful handling of the LMSs during stretching for culture and recordings (44). Additionally, as the slicing progresses from endocardium to epicardium, there is increasing heterogeneity of cellular populations depending on the depth of the LV block. This may include increased variability and can be accounted for by the randomization of the LMSs. Moreover, even though the LMS platform retains the structure of microvessels, they are not functional as they are not perfused by blood and oxygen throughout (10). Another limitation includes the difficulty in identifying the appropriate O_2_ levels to account for lack of blood flow across vessels and the absence of hemoglobin to deliver O_2_ to the tissue. In fact, in the LMS, oxygenation is achieved through diffusion. To account for this variability, the use of O_2_ electrodes is recommended for the monitoring of the O_2_ concentration real-time, and it is worth considering in hypoxia studies.

## 6 Isolation and characterisation of cardiac sEVs from LMS

### 6.1 Isolation of sEVs from LMS

The isolation method of sEVs should be determined based on their source, biophysical and biochemical properties, specificity, and desired yield. The most widely used isolation and separation methods include differential ultracentrifugation, density gradient centrifugation, size exclusion chromatography (SEC), precipitation, ultrafiltration, label-free microfluidics, immune-precipitation and affinity precipitation (20). Each of these methods offers different advantages and limitations, associated with the yield, heterogeneity, and purity of the obtained sEVs. For example, precipitation and ultracentrifugation are associated with high yield, however they isolate a mixture of extracellular particles, reducing the specificity of the EVs obtained. Similarly, differential ultracentrifugation isolates EVs depending on their size, but co-isolates non-vesicular extracellular particles. Hence, it is suggested to combine different methods to exploit the advantages of multiple techniques (11, 43). The combination of ultrafiltration with size exclusion chromatography (SEC) achieves the efficient isolation of EVs from conditioned media. It has been previously demonstrated that the combination of ultrafiltration and SEC for the isolation of EVs from culture media achieves a similar or even higher yield and purity compared to ultracentrifugation (55, 56).

Following preparation and culture of LMS, the isolation of cardiac sEVs would be achieved from the conditioned medium, in which the LMSs are cultured. The ultrafiltration strategy involves the utilization of membranes that separate the sEVs based on their molecular weight cut-off (MWCO), which is estimated to be 10–100 kDa for sEVs (57, 58). The centrifugal concentrators contain a 10,000 kDa MWCO filtration membrane that, following centrifugation reduces the final volume of the media to less than 1 ml (59).

The concentrated media then undergoes SEC. SEC utilizes polymers to create a porous stationary phase in a chromatographic column, where sEVs are separated in different path lengths according to their size. The principle is that the larger particles pass through the column first, while the smaller particles are trapped in the pores, and therefore eluted in the later fractions. The principle of the isolation techniques is that they separate smaller from larger EVs. Thus, depending on the desired size and type of EV, columns of the appropriate recovery range should be utilized (20). For the collection of sEVs, the initial fractions of the eluate are discarded, while the last fractions are the sEV-enriched. Multiple studies have used SEC to isolate cardiac sEVs from the culture media of human iPSC-CMs and cardiac fibroblasts. Recently, a study isolating sEVs from the conditioned media of human cardiac fibroblasts used ultrafiltration, followed by SEC, to achieve a high yield of cardiac sEVs (55). The combination of these two techniques achieves high sEV yield, integrity, and specificity in a short time and with limited equipment required (11, 20, 56, 60).

### 6.2 Characterisation of isolated sEVs from LMS

**Figure 4:**
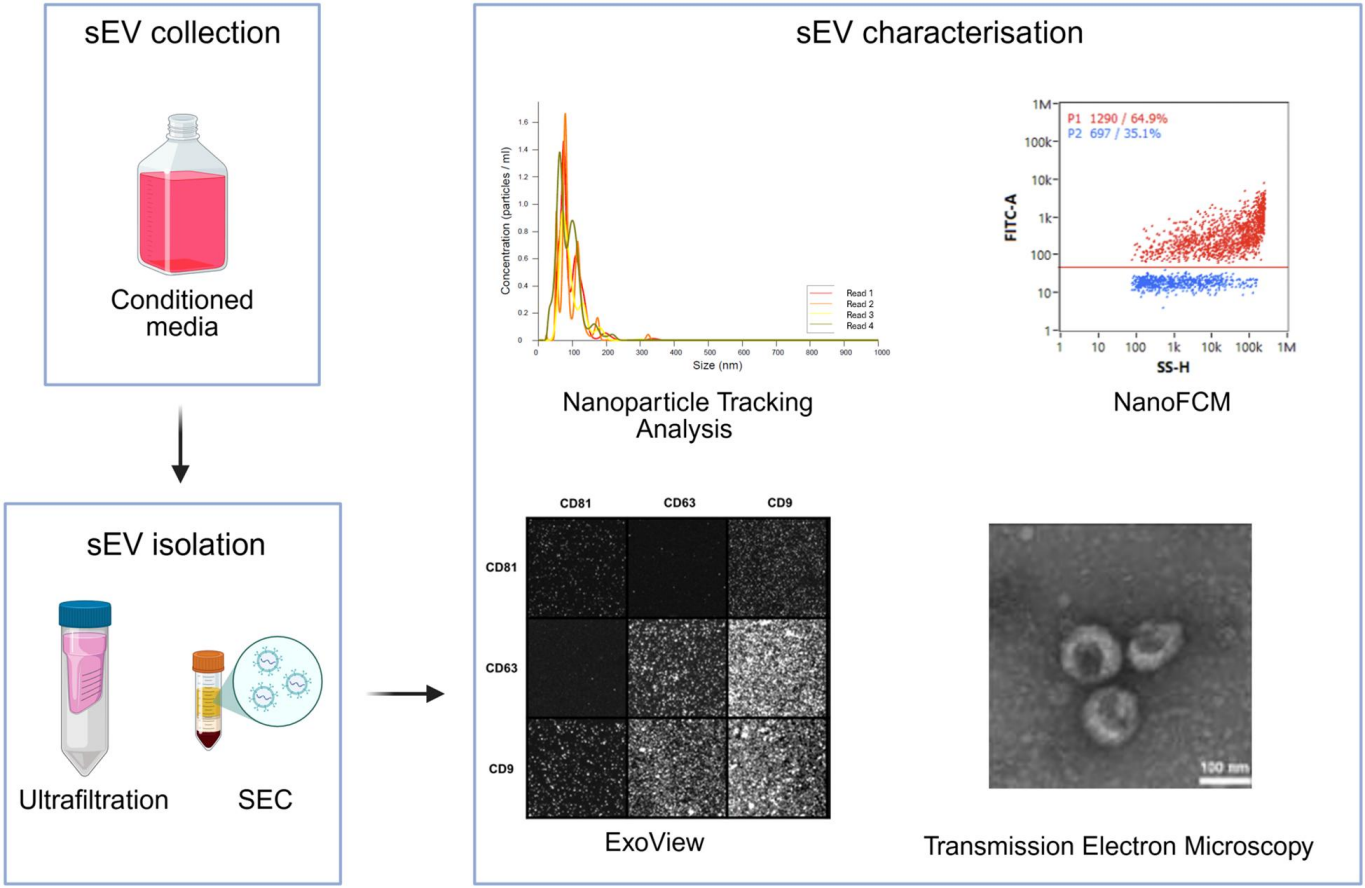
Overview of sEV isolation and characterisation methods. The LMS conditioned media is collected from the culture chamber. The sEV isolation is achieved via ultrafiltration and SEC. The isolated sEVs are characterised based on quantity and s size distribution, marker expression, and shape. Representative images of the output of NTA, NanoFCM, ExoView, and images of sEVs visualized under Transmission Electron Microscopy. “ExoView” reproduced with permission from “Representative ExoView images of indicated immunophenotypically captured and detected Serum 10 k xg EVs” and “Transmission Electron Microscopy” adapted with permission from “Quality control of EVs utilised for comparative NTA device analysis” by Daniel Bachurski, Maximiliane Schuldner, Phuong-Hien Nguyen, Alexandra Malz, Katrin S Reiners, Patricia C Grenzi, Felix Babatz, Astrid C Schauss, Hinrich P Hansen, Michael Hallek and Elke Pogge von Strandmann, licensed under CC BY-NC 4.0.

According to the MISEV, the established sEV characterisation parameters include quantity, size, sEV marker expression, and shape (11, 43), as illustrated in Figure 4. These parameters ensure that the nanoparticles collected are of sEV identity.

#### 6.2.1 Nanoparticle tracking analysis (NTA)

Nanoparticle Tracking Analysis (NTA) is a widely used technique for the characterisation of sEVs. NTA tracks average concentration, average size, and particle-size distribution of nanoparticles. NTA uses the Nanosight NS300 (Malvern Instruments) to quantify concentration and size of nanoparticles ranging from 30 to 1,000 nm. The sEVs are irradiated with a laser beam and visualised by the light scattered. This technique also provides the fluorescent mode, which allows detection of individual fluorescently labelled nanoparticles (61). The instrument is equipped with three light sources of different wavelengths (450 nm, 520 nm, and 635 nm). The NTA software applies the Stokes-Einstein equation to measure the size of the particles (62). It achieves that by tracking the Brownian motion, which is defined as the movement caused by the random collisions of nanoparticles that are dispersed in a liquid. Specifically, the presence of sEVs in a sample is calculated using the equation *D* = *k*_B_*T*/6π*ηr*, where *D* is the diffusion coefficient, *k*_B_ is the Boltzmann constant, *T* is the absolute temperature, and *η* is the dynamic viscosity of the liquid, to determine *r*, which is the radius of the nanoparticle. For the acquisition of recordings, sEVs need to be diluted accordingly, to preserve osmolality of the sEVs and ensure accurate detection and quantification, according to the manufacturer (63). The NTA can been used for EV samples derived from various sources, including plasma, urine, and cardiac cells in culture (27, 64, 65).

#### 6.2.2 Nano flow cytometry

Nano flow cytometry is another standardised method for EV quantification and size distribution analysis. The NanoFCM Flow NanoAnalyzer is a nano flow cytometer that uses light scattering to measure concentration and size distribution of nanoparticles ranging from 7 to 1,000 nm in size at a single nanoparticle level. The system also offers the option of fluorescence labeling, as it is equipped with lasers of multiple wavelengths. It is a great tool to identify specific sEV populations with the use of tetraspanin proteins, and with other markers depending on their origin. For example, it has been recently established that CD172a is a cardiac sEV-specific marker (34). This offers an additional characterisation step, allowing the quantification of sEV-sized particles that are positive for cardiac sEV markers.

Due to its high sensitivity, many attempts of optimisation are required to determine the concentration of sEVs that allow their optimal staining, as well as their detection and accurate measurement when using the NanoFCM Flow NanoAnalyzer. The optimisation process should take into account several parameters, including sEV size, concentration, chemical attributes, and markers tested. For the staining, it is recommended to test different antibody concentrations, sEV concentrations, incubation timings, and incubation temperatures. Following staining, the sample should be washed to remove free dye. Once optimised, the sample dilution used for NanoFCM Flow NanoAnalyzer recordings is also important.

This has been performed for sEVs isolated from various biofluids, cultured cells, and cells isolated from tissue (66–68). NTA and nano flow cytometry individually provide high-sensitivity readouts, however their accuracy can be influenced by parameters including sample heterogeneity, source, and isolation method (69). Thus, the combination of NTA and nano flow cytometry eliminates or reduces the limitations associated with each method individually (70).

#### 6.2.3 Exoview

The ExoView R100 is a system that is utilized to visualize, quantify, and analyze the expression of sEV markers at a single-particle level. It uses chips that contain antibody capture spots which, following the immunofluorescent staining of sEVs, indicate the presence and quantity of the tetraspanin markers attached to the chip. This system uses an interferometric reflectance imaging sensor, which achieves high resolution images to detect and count individual sEVs bound on the chip, measure their size, and quantify marker expression (71).

The ExoView has been used extensively for the characterization of EVs isolated from tissues including cardiac, foreskin, and lung fibroblasts, neuronal stem cells, but also from fluids, including plasma and bronchoalveolar lavage fluid (72–75).

#### 6.2.4 Transmission electron microscopy

Transmission electron microscopy (TEM) is used as another common step of sEV characterisation. This is particularly important for the identification of the characteristic shape of sEVs, while also enabling the evaluation of the presence of sEV markers (43). It is a widely used method for the visualization and characterization of nanoparticles, as it offers the acquisition of higher magnification than the light microscope (76). TEM has been performed for the visualization of sEVs isolated from the media of cultured CMs of adult mouse hearts, but also from human plasma (66, 77). However, it is a high-cost technique and requires expertise.

### 6.3 Application of sEVs on LMS

**Figure 5:**
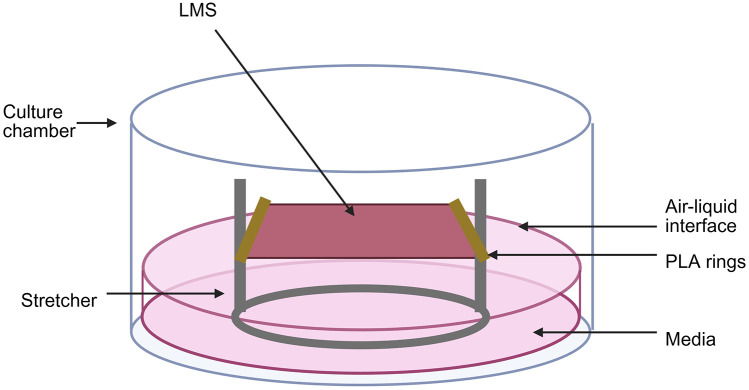
Setup of the air-liquid interface of sEVs treatment on LMS. Media is removed from the culture chamber to expose the top surface of the LMS to air. The sEVs are applied on the surface for their absorption by the LMS. Electrical stimulation, media circulation, and oxygenation are interrupted. Following absorption, media is added to achieve the desired volume and electrical stimulation, media circulation, and oxygenation are resumed.

Following quantification and characterisation of LMS-derived sEVs, it is important to investigate their mechanistic effect on LMS. This can be achieved by the application of sEVs on LMS. The sEVs are absorbed by the LMS and can influence them, according to the stage of disease they were collected at, as well as the health state of the LMS—healthy or diseased. A potential technique for the application of sEVs on LMS would be their administration on the air-liquid interface of the LMS (Figure 5). Specifically, media can be removed to expose the top surface of the LMS to air, while the bottom surface will still be in contact with the media. The volume containing the desired sEV concentration can be applied on the LMS. For this stage, dose-response experiments of sEVs on LMS are recommended, for the determination of the optimal sEV concentration. Electrical stimulation, media circulation, and oxygenation should be interrupted, to avoid dispersion of the sEVs in the media. Following absorption, the media can be added on the chamber and electrical stimulation, media circulation, and oxygenation can be resumed.

## 7 Investigation of functional, structural, and biochemical effects of sEVs on LMS

### 7.1 Investigation of functional effects of sEVs on LMS

**Figure 6:**
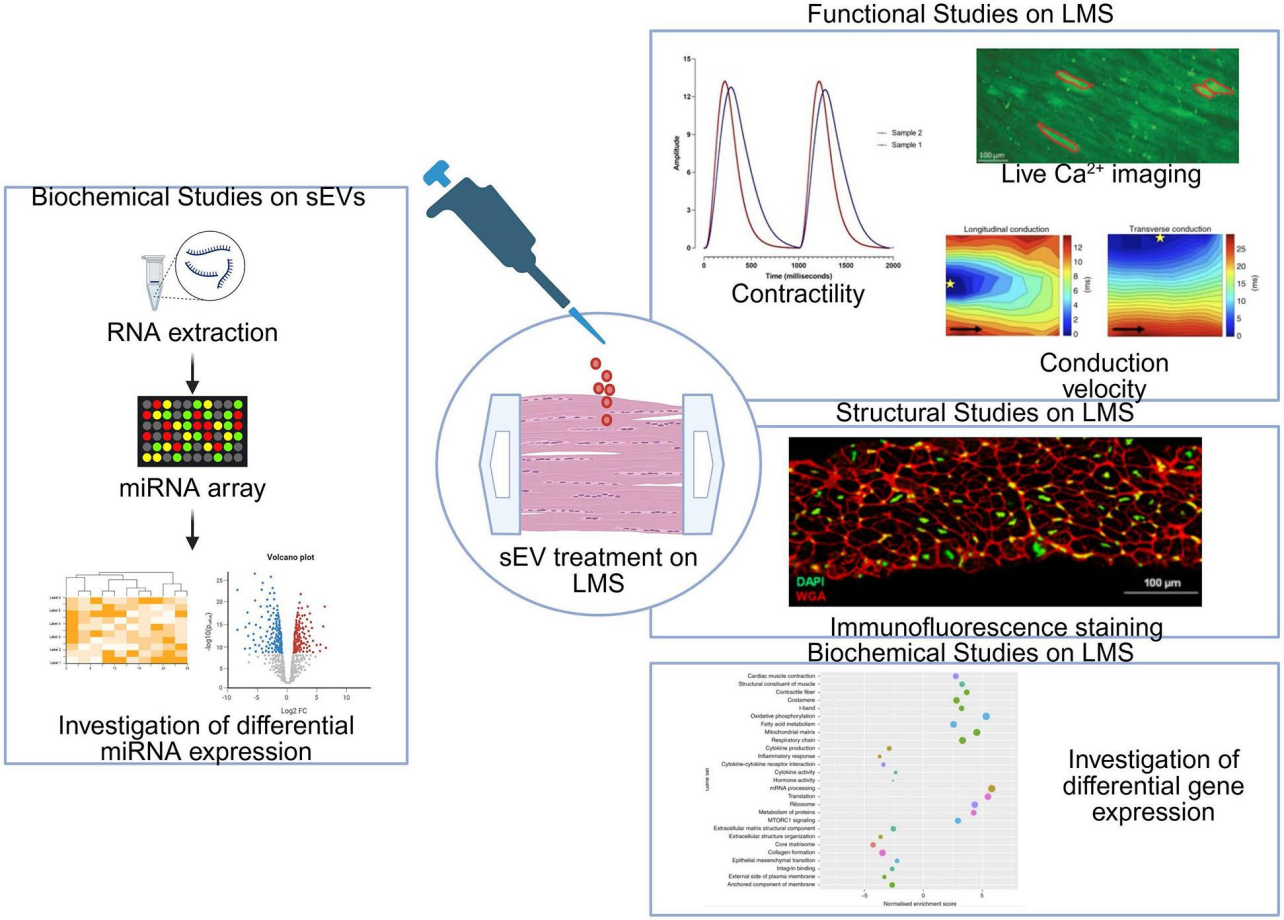
Overview of functional, structural, and biochemical studies on LMS treated with sEVs. The investigation of differential miRNA expression on sEVs can be performed by miRNA arrays. Following the treatment of LMS with cardiac sEVs, functional studies (contractility, live Ca^2+^ imaging, and conduction velocity), structural studies, and biochemical studies can be carried out on the LMS. Contractility recording adapted with permission from “Contractility recordings and representative confocal images of living myocardial slice” by Filippo Perbellini and Thomas Thum, licensed under CC BY-NC. Live Ca^2+^ adapted with permission from “Viability in freshly sliced LMS and after 24h of culture” by Eef Dries, Ifigeneia Bardi, Raquel Nunez-Toldra, Bram Meijlink and Cesare M. Terracciano, licensed under CC BY 4.0. Conduction velocity reproduced with permission from “Conduction, connexins, β-adrenergic stimulation and arrhythmogenesis in rat myocardial slices cultured for 24 h. a, b Representative images of longitudinal and transverse conduction maps of a 0 h rat myocardial slice. Black arrows show myocardial fibre direction. Yellow star = location of the stimulating electrode. Area of conduction map: 4.9 × 4.9 cm” by Samuel A. Watson, James Duff, Ifigeneia Bardi, Magdalena Zabielska, Santosh S. Atanur, Richard J. Jabbour, André Simon, Alejandra Tomas, Ryszard T. Smolenski, Sian E. Harding, Filippo Perbellini and Cesare M. Terracciano, licensed under CC BY 4.0. Immunofluorescence staining adapted with permission from “Imaging LMS” by S. A. Watson, A. Dendorfer, T. Thum and F. Perbellini, licensed under under CC BY 4.0. Investigation of differential gene expression adapted with permission from “Electromechanical regulation of gene expression in rat myocardial slices cultured for 24 h” by Samuel A. Watson, James Duff, Ifigeneia Bardi, Magdalena Zabielska, Santosh S. Atanur, Richard J. Jabbour, André Simon, Alejandra Tomas, Ryszard T. Smolenski, Sian E. Harding, Filippo Perbellini and Cesare M. Terracciano, licensed under CC BY 4.0.

The set of experiments that can be performed in terms of function, structure, and molecular profile of LMSs following sEV treatment are summarized in Figure 6. Functional studies are essential to understand the health state of the tissue and to investigate the effect of disease. Function can be evaluated by looking at contraction kinetics, live Ca^2+^ imaging, and conduction velocity. The investigation of the functional properties of LMS has been described previously but has not been performed on human LMS that have been treated with LMS-derived cardiac sEVs. However, the protocols and principles remain the same.

#### 7.1.1 Contractility studies

Functional studies for the contractility and kinetics evaluation of the LMSs are performed using the force transducer (FT) apparatus. The FT setup in our laboratory has been updated to provide biomimetic conditions, while allowing real-time measurements of functional parameters (10). The FT is wired to an amplifier that conveys the force signal to the transducer for processing.

From the force transient, parameters such as passive force, which measures the diastolic force generated upon stretch, and the active force, which expresses the maximum systolic force generated upon systole can be extrapolated. These values are normalised by the cross-sectional area of each LMS. Kinetics parameters include the time to peak (TTP), time to 50% and 90% decay, and the decay rate constant (tau) (10, 44).

The recording of these parameters is measured at multiple stretches of the sarcomere, by manipulating the length of the LMS. It is particularly interesting to investigate the functional changes under different sarcomere lengths, as according to the Frank-Starling Law, increasing the stretch of the sarcomere in healthy myocytes within physiological ranges increases the force they generate upon contraction (80).

Upon the start of the experiment, the slack length of the LMS is measured, and is gradually stretched horizontally to achieve different lengths throughout the recording. Specifically, the functional parameters of the LMS are recorded and measured at unloaded, 5%, 10%, 15%, 20%, 22% (physiological), 25%, and 30% stretch (10, 44).

Functional studies of LMS have been performed extensively in our laboratory for several disease models. Rat LMS have been used to evaluate the functional effects of the inhibition of calcium/calmodulin-dependent protein kinase II in cryo-injured LMS (52). Another study has investigated the effect of different sarcomere lengths following 24 h in culture on the contraction amplitude of rat LMS. In the same study, human failing hearts were used for the generation of LMS to evaluate the effect of electromechanical stimulation on their function. It was reported that the optimal preload applied in culture is equivalent to a sarcomere length of 2.2 μm (78). From these studies, it is evident that the LMS model can be used for the performance of contractility experiments, while providing an insight for many models of disease.

#### 7.1.2 Live Ca^2+^ imaging studies

The evaluation of live Ca^2+^ imaging provides valuable information regarding the electrophysiological capacities of the LMS in homeostasis and injury, including Ca^2+^ transient kinetics and intracellular Ca^2+^ concentrations. These parameters can be affected by disease, thus providing an insight into the effects of CVD progression. This is achieved with the use of fluorescent indicators that are loaded in the cells and upon illumination, will fluoresce with every contractile cycle of the LMS.

Common fluorescent probes include Fluo-4AM and Fluo-8AM, which have been previously applied in rat LMSs (52). Other Ca^2+^ indicators include Rhod-AM, which has been used for swine, rabbit, and human models (81–83). It is important to note that Rhoad2AM can also detect mitochondrial Ca^2+^ activity, thus Fluo-4AM or Fluo-8AM might be more reliable (84). Also, during the incubation with the fluorescent indicator, the LMS should remain on the stretcher to maintain the physiological load. It is important to use blebbistatin or 2,3-butanedione monoxime to inhibit the activity of myosin (79), thus allowing the successful loading of the indicator.

Recently, Ca^2+^ kinetics were evaluated in a human LMS model. Specifically, the researchers were interested in the electrophysiological changes that occur in the LMS model following long-term culture of approximately 10 days. Thus, they used Rhoad2AM to evaluate the Ca^2+^ handling properties of the LMS and were able to identify an improved function of the LMS following culture, confirmed by the increased Ca^2+^ transient amplitude and reduced duration. This shows that the investigation of Ca^2+^ dynamics is possible with human LMS (83). Even though the effects of the application of LMS-derived cardiac sEVs on human LMSs on the Ca^2+^ activity have not been previously evaluated, the protocol should be the same and can be applied following sEV application and desired culture duration.

#### 7.1.3 Conduction velocity studies

Conduction velocity (CV) studies estimate the speed at which the electrical impulse travels through the cardiac tissue, to induce an action potential. The investigation of CV is essential, as it has shown to be influenced in CVD (85). The LMS model can be used as a platform to study CV by its attachment on a multi-electrode array (MEA) system. CV can be measured longitudinally or transversally to myofiber orientation. In our laboratory, conduction velocity studies have been optimized for rat and human LMSs (78, 86). Briefly, the MEA used is the MEA1060 (Multi Channel Systems), which contains 60 microelectrodes in an 8 × 8 arrangement. The LMS is attached in the center of the system, in combination with a holder to improve its interaction with the electrodes. During the recording, the sample is perfused with oxygenated Tyrode solution and maintained at 37°C. Electrical stimulation at a frequency of 1 Hz from different MEA microelectrodes and with the lowest voltage and duration to achieve the threshold allows field potential recording for CV evaluation. Arrhythmia and spontaneous field potentials can also be studied. As with contractility and Ca^2+^ studies, this protocol can be repeated following sEV application.

### 7.2 Investigation of structural effects of sEVs on LMS

#### 7.2.1 Immunostaining of LMS treated with sEVs

Prior to application on LMS, sEVs can be stained to offer the option of visualization on the LMS for structural studies. There are staining kits that have been widely used. For LMS-derived cardiac sEVs, the ExoGlow (System Biosciences) kit has been successful. It uses a fluorescent dye that stains the lipid bilayer of sEVs and has been used extensively for EV research (87).

For investigation of sEV uptake and biodistribution on the LMS, the cellular components of the tissue should be stained with appropriate cell-specific membrane dyes. The structural effect of cardiac-derived sEVs on the LMS can be evaluated via the immunofluorescence staining of multiple cell types. This will allow measurement of several parameters that can be affected by the pathways influenced by sEV content. Recommendations include WGA for evaluation of hypertrophy, collagen and vimentin for fibrosis and stromal cell proliferation, connexin-43 for gap junction formation between cells, isolectin B-4 and CD31 for angiogenesis, TUNEL for apoptosis, a-actinin for CM characterisation and measurement of sarcomere length, and any cellular processes of interest. Quantification of these proteins can be performed by protein extraction and Western blot, following optimised protocols for LMS (10, 44, 51, 52).

Stained sEVs, with combination of cellular staining, allows the investigation of sEV biodistribution on the LMS. Biodistribution evaluation is of particular interest in sEV applications, as it can inform about the tropism of the sEVs on the LMS. Specifically, quantification of sEVs within different cell types can indicate their preferential uptake by specific cell types in a multicellular environment and can inform about their uptake at specific timepoints of disease.

### 7.3 Investigation of molecular effects of sEVs on LMS

#### 7.3.1 miRNA profiling and bioinformatics analysis

The miRNA content of cardiac sEVs is of particular interest in the CVD field, as miRNAs play a major role in the regulation of gene expression during health and disease. miRNAs are post-transcriptional regulators of gene expression, and their levels change dynamically in different stages of CVD (12). The miRNA content of sEVs reflects the state of the donor cell and gives an insight into the molecular changes that happen to cells in health and disease. Individual miRNA are potent mediators of disease progression, and identifying specific sEV miRNAs can give insights into disease mechanisms (88). The LMS model plays a key role because it gives us the ability to isolate myocardium-specific sEVs of adult tissue and with the contribution of all different cardiac cell types (44). The application of sEVs derived from LMS can provide a deeper understanding of their functional and structural effects on different CVDs and of their role in cardiac intercellular communication.

To investigate the cargo of sEVs, the RNA should be extracted and applied on miRNA arrays. miRNA arrays work as standard polymerase chain reaction (PCR) panels that allow the profiling of hundreds of miRNAs from small RNA quantities and allow the quantification of their relative expression in a given sample (89). Following analysis of the miRNA array, the upregulated and downregulated miRNAs can be validated by quantitative PCR (qPCR) and inserted into bioinformatics databases to identify potential target genes for downstream analysis (90).

Several studies have investigated the miRNA content of cardiac sEVs from different sources, with the aim to uncover differential miRNA expression. A recent study investigated the miRNA profile of sEVs derived from induced pluripotent stem cell-derived cardiomyocytes (iPSC-CMs) from dilated cardiomyopathy patients. The iPSCs were differentiated into CMs in culture and the secreted sEVs were isolated from the conditioned media. miRNA profiling confirmed the upregulation of miR-218-5p, which is associated with increased fibrosis through the activation of TGF-β signaling pathway (91). In another study, the RNA extraction of sEVs secreted from cultured adult mouse CMs allowed for the profiling and identification of 1,520 miRNA, of which 423 could be associated with a biological network (77). Another study performed profiling of EV-derived miRNA from the plasma of patients with aortic valve stenosis, to investigate the mechanism behind valvular cell calcification. Circulating sEV miRNAs were profiled using a miRNA array that identified the presence of 254 miRNAs, of which the 51 were differentially expressed between aortic valve stenosis and healthy patients. The upregulated miR-455-3p and downregulated miR-103a-3p were linked with pathways that can influence the calcification pathway in aortic valve stenonis patients (92). These studies show that the identification of changes in specific miRNAs can lead to their linking with specific pathways, thus explaining the progression of disease phenotypes.

A recent study compared the miRNA profile of cardiac EVs secreted from murine healthy and failing hearts, using a bioinformatic approach. Following validation with miRNA array and qPCR, 7 upregulated and 3 downregulated miRNAs were found. Bioinformatics analysis confirmed that the unregulated miRNAs were involved in cell cycle, signal transduction, and apoptosis pathways, while the downregulated were associated with cell cycle, apoptosis, and transcription and translation processes (93). This is another example of the importance of linking miRNA changes to genetic pathways and biological processes, to explain the changes observed in disease.

LMS are an unprecedented tool for the study of sEVs in cardiovascular physiology and disease, offering a wide range of potential applications. One of the potential applications with the LMS model would be the investigation of the gene expression profile of the cells within the LMS. Specifically, the isolation of different cell types of the LMS for the performance of bulk sequencing combined with bioinformatics analyses can confirm whether the miRNA changes have an effect on the gene expression levels of their target genes (93). This information can explain the structural and functional changes during disease progression. The LMS model offers unique applications for studies using sEVs. Firstly, it can be used for investigating the tropism of sEVs and potentially identifying their preferential site of uptake in the heart. Secondly, it can be used to track which specific cell type in this multicellular platform releases the most sEVs in health and disease. Future advancements can enhance delivery through engineering them with specific receptors or ligands, based on their native characteristics (94).

These studies highlight that investigating differential expression of miRNAs is a critical step for gaining a deeper understanding of the mechanism driving CVD progression. Transitioning this research from animal or iPSC-derived models to human LMS models is a significant advancement, as it enhances the relevance and translational potential of the findings to potentially improve the prognosis, prevention, or treatment of CVD.

## 8 Clinical and translational implications

It is important to highlight the growing significance of sEV research and its potential impact on the cardiovascular field. It is established that sEVs regulate intercellular communication on homeostasis and disease (19). This role makes them important tools for the diagnosis, treatment, and prevention of disease. Firstly, due to their altered levels in disease, circulating sEVs can act as biomarkers for diagnosis and prognosis. Their high potential as biomarkers is associated with their stability upon isolation and their presence in virtually any body fluid. Moreover, the content of sEVs portrays health state of the cells that secrete them and can enter circulation, thus enabling their detection (95). Additionally, the content of sEVs is protected by their membrane and is not degraded or dispersed (88). An example of an established CVD biomarker is cardiac troponin, which is elevated following acute MI. Similar trends have been observed with cardiac miRNAs. Plasma samples from patients that had undergone CABG surgery exhibited an increase in miR-499 levels, making it a biomarker of perioperative MI (96). A cohort analysis with data from 2,500 patients identified miR-499 and miR-208b to be displaying comparable specificity to that of cardiac troponin T as a biomarker of MI (97). The LMS model can be used to validate these findings, using adult human tissue in order to identify novel biomarkers of CVD with a higher translational potential.

Advancements in the synthetic biology and bioengineering field allow the use of sEVs as therapeutics' delivery tools (98, 99). In this context, the sEVs can be engineered for targeted drug delivery (7). Specifically, tools can be used for the loading of sEVs with bioactive molecules that can be delivered to the recipient cells, functioning as a therapeutic (100). Advances in bioengineering also allow addition of cell-specific ligands to target specific cells (94). Another advancement is the use of Y-box proteins to sort miRNAs into sEVs in cells (101). However, these techniques require refinement prior to translation, as there are risks associated with disturbing sEV structure and content, thus affecting their ability to travel through the extracellular environment and to the target cell. Transfection has been performed for the loading of sEVs secreted from cardiac stromal cells with miR-21-5p—a known downregulated miRNA in heart failure—to stimulate the regeneration of the damaged myocardium (102). Another study used electroporation to load sEVs with anti-inflammatory agents, achieving a 20% loading efficiency and observing a reduction in inflammation in a model of atherosclerosis (103).

## 9 Conclusion

CVD is the leading cause of death globally. The high complexity of CVD combined with the lack of appropriate experimental models has prevented the understanding of the mechanisms that contribute to its detrimental effects. This has led to the lack of efficient preventative methods and effective treatment options (1, 3).

The sEV field has seen significant advances in recent years. The increasing understanding of their participation in cellular processes of homeostasis and disease has become more evident in multiple fields (19). In the cardiovascular field, sEV miRNA content has been confirmed to influence cellular function, structure, and molecular profile, affecting the progression of CVD (91, 97, 104). The mechanism behind the concentration and content changes of sEVs in response to external stimuli—including disease—have not been fully uncovered. This is related to the lack of relevant experimental models that allow isolation of a pure population of cardiac-specific sEVs, as well as the inaccuracies of the instruments for the detection and measurement of nano-sized particles.

This review acts as a guide to introduce LMS-derived cardiac sEVs that are of pure cardiac origin and secreted by the multicellular environment that composes the adult human myocardium. This is a significant step forward compared to studies that isolate sEVs from body fluids, where the source of sEVs is difficult to determine. The multicellular LMS platform offers a more efficient way to isolate pure cardiac sEVs, without the involvement of other organs (44). Meanwhile, the electrical stimulation, oxygenation, and mechanical preload conditions, combined with the circulation of media rich in hormones and vitamins, provide a highly physiological environment for the LMS. This environment could act as a confounder on the quantity of sEVs secreted and on their content, which could potentially show great differences in comparison with previously reported minimalistic models. The procedures mentioned above can allow the isolation of sEVs that are produced by cardiac tissue during health and disease conditions, opening avenues for further investigation on their functional, structural, and molecular influence on the myocardium. In conclusion, the growing sEV field shows high potential in the development of the CVD field, offering new strategies for diagnosis and treatment of cardiac disease. The combination of cardiac sEVs with the LMS model can offer a more relevant and translational model for the understanding of the mechanistic role of sEVs in the functional, structural, and biochemical effects of different CVDs.
